# SERS‐Based Assessment of DNA Methylation for the Evaluation of Measurable Residual Disease in Acute Promyelocytic Leukaemia

**DOI:** 10.1111/jcmm.70244

**Published:** 2025-03-05

**Authors:** Anamaria Bancos, Stefania D. Iancu, Vlad Moisoiu, Alexandra Ghiaur, Adrian Bogdan Tigu, Cristina Buran, Georgiana Ion, Camelia Stancioaica, Bogdan Ionescu, Mihaela Dragomir, Codruta F. Buldus, Diana Cenariu, Madalina Nistor, David Kegyes, Diana Gulei, Nicolae Leopold, Daniel Coriu, Ciprian Tomuleasa

**Affiliations:** ^1^ Department of Hematology—Medfuture Research Center for Advances Medicine Iuliu Hatieganu University of Medicine and Pharmacy, Cluj Napoca Romania; ^2^ Faculty of Physics Babeș‐Bolyai University Cluj‐Napoca Romania; ^3^ Department of Leukemia Fundeni Clinical Institute Bucharest Romania; ^4^ Medfuture Research Center for Advances Medicine Iuliu Hatieganu University of Medicine and Pharmacy, Cluj Napoca Bucharest Romania; ^5^ Faculty of Physical Education and Sport Babeș‐Bolyai University Cluj‐Napoca Romania

**Keywords:** acute promyelocytic leukaemia, minimal residual disease, SERS

## Abstract

Acute promyelocytic leukaemia (APL) is a type of acute myeloid leukaemia characterised by the reciprocal translocation t(15;17), which offers a unique possibility for measurable residual disease (MRD) monitoring by PCR amplification of the *PML‐RARA* transcripts. Surface‐enhanced Raman scattering (SERS) is a laser molecular spectroscopy technique that allows the rapid analysis of changes in the DNA methylation pattern associated with malignant transformation. In this study involving 49 DNA samples from bone marrow aspirations from patients with APL, we showed that the DNA from MRD‐positive samples exhibited lower SERS intensities of the band at 730 cm^−1^ attributed to adenine. Next, we used the scores derived from principal component analysis (PCA) as input data for machine learning algorithms trained to categorise SERS spectra based on MRD status. The results showed that the highest classification accuracy was achieved by logistic regression, yielding an area under the ROC curve (AUROC) of 0.90. In addition, PCA showed that samples corresponding to patients with FLT3‐ITD mutations had a tendency for unsupervised clustering irrespective of MRD status. These results suggest that SERS analysis of DNA represents a promising method for the MRD monitoring of APL patients. Future validation of these findings in large prospective studies is warranted.

## 1 Introduction

Acute promyelocytic leukaemia (APL) is a unique biological and clinical variant of acute myeloid leukaemia, accounting for up to 10%–15% of the newly diagnosed acute myeloid leukaemias in adults [1, 2, 3]. The molecular hallmark of APL is a balanced reciprocal chromosomal translocation, t(15;17), which results in a chimeric *PML‐RARA* fusion gene that offers a unique opportunity for measurable residual disease (MRD) monitoring via quantitative real‐time reverse‐transcription PCR (qRT‐PCR) of the *PML‐RARA* transcripts [4]. Along with other molecular markers such as the internal tandem duplication mutations in the *FLT3* (*FLT3‐ITD*), which is an actionable vulnerability bearing a negative prognostic significance [5], the MRD has transformed the management of the patients with APL [4].

Surface‐enhanced Raman scattering (SERS) is a method to amplify the Raman signal of molecules that are adsorbed onto the SERS substrates such as gold or silver nanoparticles [6]. Several lines of evidence showed that the cancer‐associated changes in the DNA methylation pattern promote the preferential assembly of DNA onto the metal gold nanoparticles [7, 8, 9], suggesting that this technique could potentially be used for the MRD monitoring of cancer.

In this study, we used SERS to analyse the DNA from the bone marrow of patients with PML, with the aim of delineating the SERS spectral features associated with MRD positivity and to benchmark SERS against qRT‐PCR, which is the gold standard for MRD detection in APL [7, 8, 9].

## 2 Materials and Methods

We analysed 49 DNA samples extracted from bone marrow aspirations from 30 patients with APL, of which 15 samples exhibited MRD positivity for the *PML‐RARA* transcript by qRT‐PCR. The demographics of the patients included in this study are shown in Table S1. The SERS amplification was performed using silver nanoparticles synthesised by reducing Ag + with hydroxylamine hydrochloride. A detailed account of the materials and methods, including the characterisation of the SERS substrate, is found in the Supporting Information (Figures S1 and S2). The study was approved by the Review Board of the Ion Chiricuta Cancer Center, Cluj‐Napoca, Romania.

## 3 Results

**FIGURE 1:**
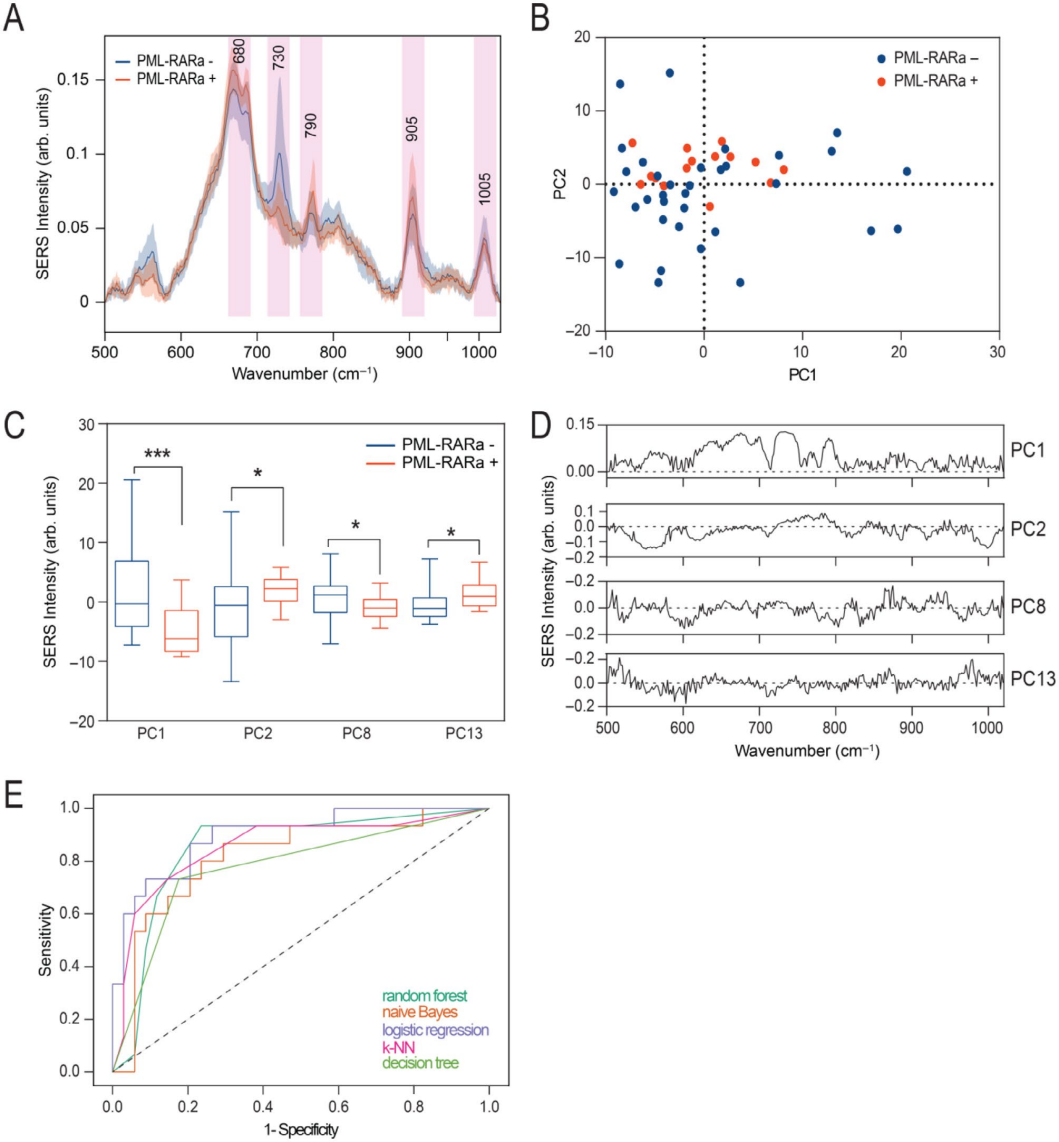
(A) Mean spectra of processed SERS spectra of genomic DNA samples from 15 acute promyelocytic leukaemia patients measurable residual disease (MRD) positive for *PML‐RARA* and 34 samples MRD negative for *PML‐RARA*; (B) Score plot depicting principal component 1 (PC1) and PC2 of SERS spectra from DNA; (C) Score values for PCs that exhibited statistically significant values between the MRD positive and MRD negative groups by Student`s t test (*p < 0.05; ***p < 0.001); (D) The loading plots for PCs that exhibited statistically significant values between the MRD positive and MRD negative groups; (E) The receiver operating characteristic curves for the discrimination between the MRD positive and MRD negative groups.

The average SERS spectra of genomic DNA from bone marrow aspirations obtained from APL patients categorised based on the presence or absence of *PML‐RARA* transcripts (MRD positivity) are shown in Figure 1A, revealing distinctive SERS bands associated with guanine (680 cm^−1^), adenine (730 cm^−1^), cytosine (790 cm^−1^), the phosphate backbone (905 cm^−1^) and 5‐methylcytosine (1005 cm^−1^) (Figure 1A). The spectral changes associated with MRD positivity exhibited a complex pattern, the most obvious change being a decrease in the SERS band at 730 cm^−1^ associated with the breathing vibrational mode of adenine.

Next, we performed PCA, which revealed a tendency for the unsupervised clustering of the SERS spectra based on the MRD status (Figure 1B). The first 20 PCs were retained for subsequent analysis, accounting for 93% of the initial variance in the dataset. Among these 20 PCs, only four (PC1, PC2, PC8 and PC13) exhibited a statistically significant difference in score distributions between samples with positive and negative MRD (Student's *t*‐test, *p* < 0.05) (Figure 1C,D).

To gain quantitative insights concerning the discrimination accuracy of SERS, the scores derived from the four PCs were used as input parameters for four machine learning algorithms trained to categorise SERS spectra based on MRD status. The results showed that the highest classification accuracy was achieved by logistic regression, yielding an area under the ROC curve (AUROC) of 0.90 (Figure 1E and Table 1). The confusion matrix for the logistic regression model, provided in Table S2, indicates that 11 samples were classified as true positives and 27 as true negatives.

**TABLE 1:** The classification accuracies yielded by machine learning algorithms trained on the SERS spectra of DNA from patients with acute promyelocytic leukaemia. The dataset was comprised of 15 DNA samples for which the qRT‐PCR showed the presence of *PML‐RARA* transcripts and 34 samples in which no *PML‐RARA* transcripts were detected. The validation of the machine learning algorithms was performed by leave‐one‐out cross‐validation. k‐NN = k‐nearest neighbours.

Model	AUROC	Accuracy	Specificity	Sensitivity
Random forest	0.82	81.6	81.3	73.3
Naïve Bayes	0.82	75.5	74.3	77.9
Logistic regression	0.90	77.6	75.2	79.1
k‐NN	0.86	83.7	70.4	83.5

Finally, we were interested to explore whether mutations in *FLT3* (*FLT3‐ITD*), which are actionable therapeutic vulnerabilities that bear a negative prognostic significance [5], influence the SERS spectra of DNA. By performing PCA only on the SERS spectra corresponding to DNA samples for which the *FLT3* mutation status was also available, we could show that there was a tendency for the *FLT3‐ITD* group to cluster together (Figure S4). However, the small number of samples in the *FLT3‐ITD* group prevented any further quantitative analysis.

## 4 Discussion

The SERS spectra of DNA associated with MRD positivity exhibited a complex pattern of changes, including a decrease in the SERS intensity of the band at 730 cm^−1^ attributed to adenine as well as less pronounced differences in other SERS bands. For the moment, the exact mechanism behind this spectral pattern is incompletely understood. In a series of studies concerning haematological malignancies, we have previously demonstrated that the recurring spectral pattern associated with DNA from malignant cells is represented by an increase in the SERS bands attributed to adenine at 730 cm^−1^ and a decrease in the intensity of the SERS band around 1005 cm^−1^ attributed to 5‐methylcytosine [8, 9]. This pattern was seen in the case of DNA extracted from peripheral blood leucocytes (buffy coat) from patients with acute myeloid leukaemia [7], from lymph node biopsies in the case of lymphomas [8] and from blood smears in the case of myeloid malignancies associated with Down syndrome [9]. Mechanistically, this pattern emerges because cancer DNA exhibits a decrease in the global levels of DNA methylation [7], resulting in a decrease in the intensity of the SERS band around 1005 cm^−1^ (attributed to 5‐methylcytosine). In parallel, the altered DNA methylation pattern changes the interaction geometry between the DNA and the metal surface [10], resulting in an increase in the SERS band at 730 cm^−1^ attributed to adenine. In contrast to the previous studies, the SERS spectra of DNA from the MRD positive group showed a decrease in the intensity of the SERS band at 730 cm^−1^, while the intensity of the SERS band at 1005 cm^−1^ was not drastically changed. One possible explanation for this discrepancy is represented by the findings reported by Schoofs et al., who showed that DNA from APL cells is not globally hypomethylated but rather hypermethylated [11]. However, there is a need for more studies to elucidate the precise molecular origin of this observation.

Next, we selected four PCs derived from the SERS spectra as input for training four machine learning algorithms. The best classification accuracy was achieved by logistic regression (AUROC of 0.90), thus demonstrating the potential of SERS as a strategy to perform MRD testing in patients with APL.

Given the prognostic and predictive significance of FLT3‐ITD, which represents an actionable vulnerability in APL that bears a negative prognostic value, we showed that the SERS spectra of DNA corresponding to cases of APL with FLT3‐ITD mutation exhibit a distinct spectral pattern, although the limited number of samples prevented a proper quantitative analysis of the classification accuracy. This finding might be explained by the fact that FLT3‐ITD is associated with higher white blood cell counts, with M3v variant morphology, and the bcr3 isoform [12], which might result in an altered spectral pattern through currently unknown mechanisms. For a more detailed discussion, see the Supporting Information (Figures S3 and S5).

## 5 Conclusion

In this study, we showed that MRD positivity is associated with a distinct SERS spectral pattern of DNA, including a decrease in the intensity of the SERS band at 730 cm^−1^ attributed to adenine. Using the SERS spectra of DNA as input for machine learning algorithms, it was possible to discriminate the samples corresponding to the MRD positive group with an AUROC of 0.90 (logistic regression), thus demonstrating the potential of SERS analysis of DNA for the MRD monitoring of APL.

## Author Contributions

**Anamaria Bancos:** formal analysis (equal), investigation (equal). **Stefania D. Iancu:** investigation (equal). **Vlad Moisoiu:** investigation (equal). **Alexandra Ghiaur:** investigation (equal). **Adrian Bogdan Tigu:** investigation (equal). **Cristina Buran:** investigation (equal). **Georgiana Ion:** investigation (equal). **Camelia Stancioaica:** investigation (equal). **Bogdan Ionescu:** investigation (equal). **Mihaela Dragomir:** investigation (equal). **Codruta F. Buldus:** investigation (equal). **Diana Cenariu:** formal analysis (equal). **Madalina Nistor:** investigation (equal). **David Kegyes:** investigation (equal). **Diana Gulei:** investigation (equal). **Nicolae Leopold:** methodology (equal), visualization (equal). **Daniel Coriu:** supervision (equal), validation (equal). **Ciprian Tomuleasa:** formal analysis (equal), funding acquisition (equal), writing – review and editing (equal).

## Ethics Statement

The study was approved by the Review Board of the Ion Chiricuta Cancer Center, Cluj‐Napoca, Romania.

## Conflicts of Interest

The authors declare no conflicts of interest.

## Supporting information


Appendix S1.

## Data Availability

All data is available upon reasonable request.
