# Supplementary material for: SERS‐Based Assessment of DNA Methylation for the Evaluation of Measurable Residual Disease in Acute Promyelocytic Leukaemia
Source: J Cell Mol Med. 2025 Mar 5;29(5):e70244. doi: 10.1111/jcmm.70244 (PMC11880916; doi:10.1111/jcmm.70244)
Supplement: Supplementary file 1 — Appendix S1. [file JCMM-29-e70244-s001.docx]

**Detailed Materials and Methods**

*Patients and DNA extraction*

The cohort included patients with APL from the Ion Chiricuta Cancer Center, Cluj-Napoca, Romania and the Department of Hematology, Fundeni Clinical Institute, Bucharest, Romania. The study was approved by the Ethics Committee of the Ion Chiricuta Cancer Center, Cluj-Napoca, Romania.

DNA was extracted from bone marrow aspirates using the PureLink Genomic DNA extraction kit (Thermo Fisher Scientific, cat no. K182002) and eluted in Ultrapure water. The DNA quality and concentration was measured by Nanodrop 2000 (Thermo Fisher Scientific). The DNA was stored at -80 °C until further use. The bone marrow aspirate samples from which the DNA was extracted were the same as the ones from which the MRD measurements were done, according to the IVD-accredited laboratory testing published by the European LeukemiaNet guidelines.

*Surface-enhanced Raman scattering*

Colloidal silver nanoparticles, synthesized through the reduction of Ag^+^ with hydroxylamine hydrochloride (hya-AgNPs) (Leopold & Lendl, 2003), served as the enhancement substrate for the SERS analysis of DNA. The colloidal hya-AgNPs were prepared by dissolving 17 mg of AgNO_3_ in 90 mL of ultrapure water under continuous stirring. Simultaneously, 17 mg of hydroxylamine hydrochloride was dissolved in 8.8 mL of ultrapure water, and 1.2 mL of NaOH 1% was added. The resulting hydroxylamine solution was added to the AgNO_3_ solution under stirring, causing an immediate color change to yellow-gray. The colloidal solution was then stored under ambient conditions. All chemical reagents were purchased from Sigma-Aldrich (Merck).

For acquiring SERS spectra, we employed an InVia Raman spectrometer (Renishaw) equipped with a frequency-doubled Nd:YAG laser emitting at 532 nm. The laser was focused on the sample through a 20X objective (Leica, NA 0.4). To maintain uniform DNA concentration, we diluted all DNA samples to a concentration of 20 ng/µL. For the SERS measurement of DNA, we mixed 5 µL of AgNPs, 5 µL of DNA, and 1 µL of Ca(NO_3_)_2_ (final Ca^2+^ concentration 5x10^-2^ M). The resulting mixture (5 µL) was placed on a microscope slide covered with aluminum foil and analyzed by SERS in liquid drop form (before the droplet dried).

*Statistical analysis*

We processed SERS spectra using the Quasar-Orange software from the Orange-Spectroscopy library (Bioinformatics Laboratory of the University of Ljubljana) (Toplak et al., 2021). We retained the spectral range of 500-1020 cm^-1^ for further analysis, subtracting the baseline using the Rubber-band function and normalizing the spectra vectorially.

Principal component analysis (PCA) was used to explore the data. The first 20 principal components (PCs) were kept for further analysis. The student’s t-test was applied to identify PCs that exhibited statistically significant differences between MRD positive and MRD negative groups. A p value < 0.05 was considered statistically significant. The score values of PCs that were statistically different between the MRD positive and MRD negative groups were used as input variables for four machine learning algorithms (Random Forest, Logistic Regression, Naïve Bayes, k-nearest neighbors). The classification model was cross-validated using leave-one-out cross-validation. The machine learning algorithms were trained using Quasar-Orange software from the Orange-Spectroscopy library.

**AgNPs characterisation**

Hya-AgNPs were characterized in terms of diameter and stability using dynamic light scattering (Zeta Sizer, Malvern Panalytical), which revealed an average diameter of 53 nm and a concentration of 6x10^10^ nanoparticles/mL. The zeta potential of hya-AgNPs was -23 mV, indicating good stability. The UV-Vis spectrum of the Hya-AgNPs is shown in Supplementary Figure 1, demonstrating the characteristics plasmonic band around 400 nm.


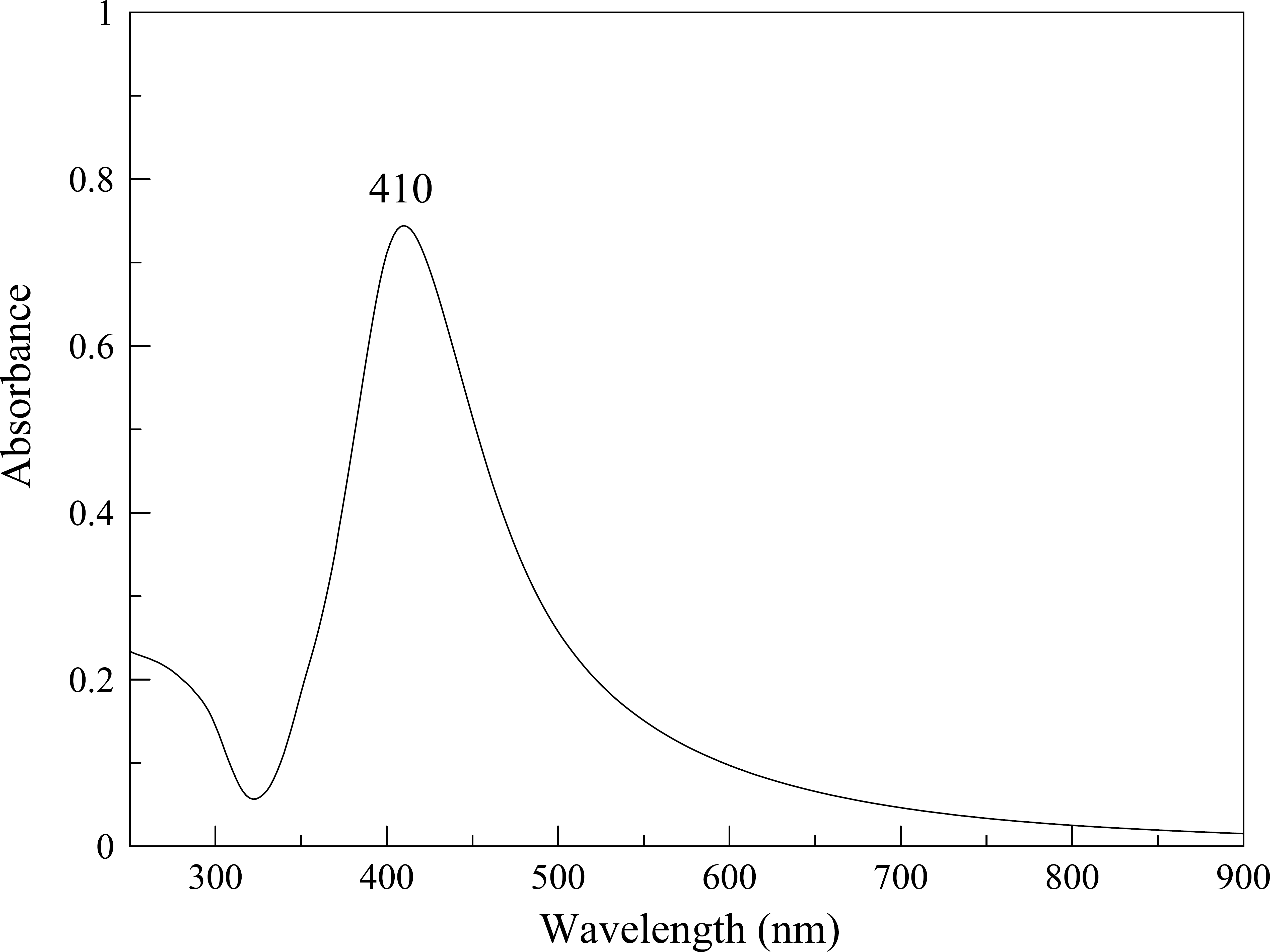


**Figure S1.** UV-Vis spectrum of the Ag nanoparticles.


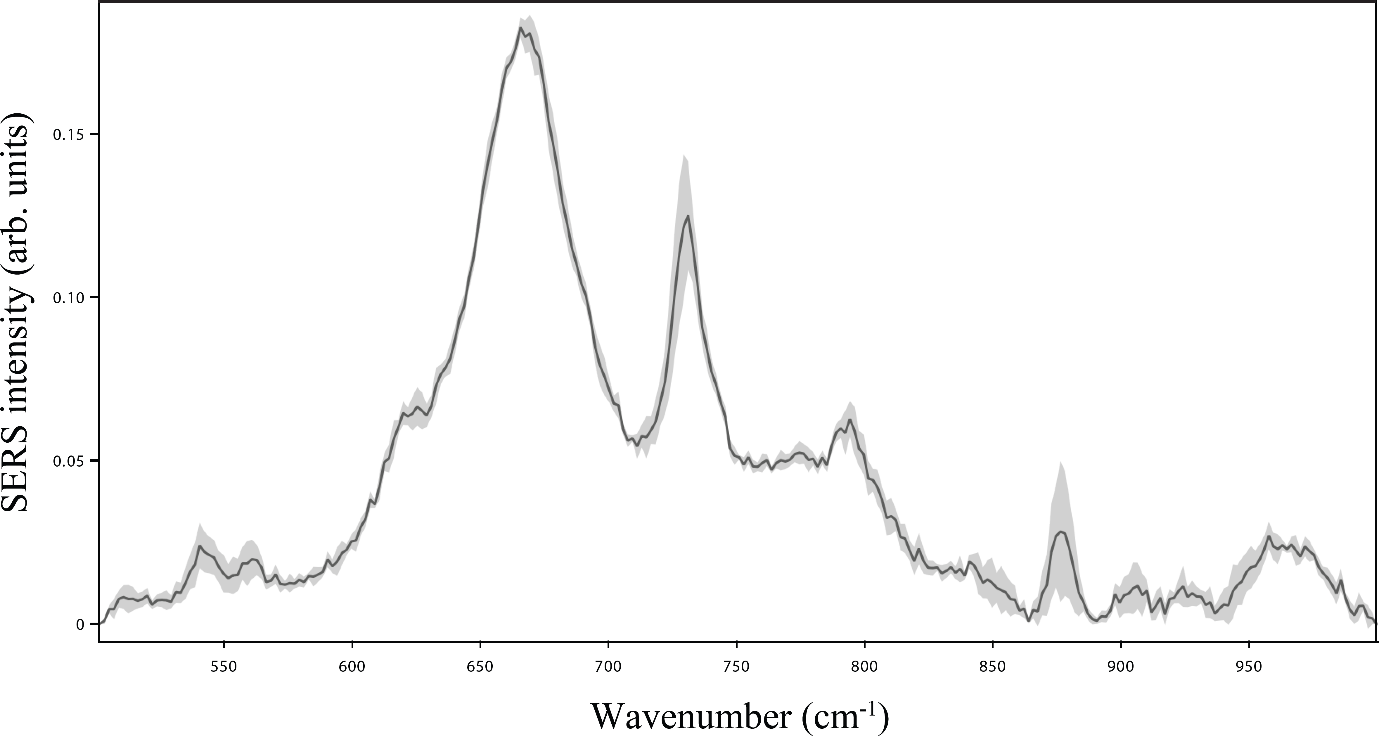
To assess the reproducibility of SERS measurements, genomic DNA extracted from the NB4 cell line was used (20 ng/mL). Four individual samples were prepared, and SERS spectra were recorded for each. The average SERS spectra, along with their standard deviation, are presented in Supplementary Figure 2. The observed variation in the SERS signal was less than 10% of the total signal.

**Figure S2.** Reproducibility of SERS spectral acquisition for genomic DNA samples. The figure shows the mean SERS spectrum and the standard deviation across four repetitions.

Additionally, we determined the detection limit of DNA using SERS. SERS spectra were recorded for varying DNA quantities, ranging from 10 ng to 500 ng (Supplementary Figure 3). No SERS signal was detected below 10 ng of DNA. A linear relationship between DNA quantity and the SERS signal was not observed. The intensity of DNA SERS bands increased with concentration up to 250 ng, but beyond this point, there was no further increase. In fact, a slight decrease in the SERS signal was observed when larger amounts of DNA were used.


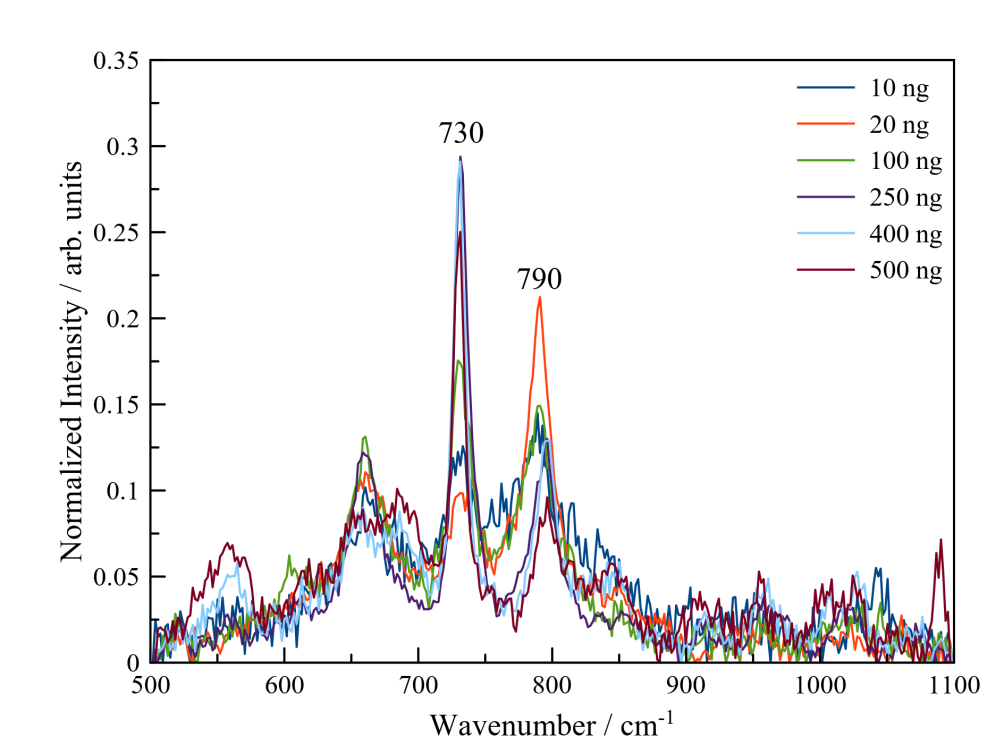


**Figure S3.** Variation in the SERS spectra of DNA across different DNA quantities in the range of 10 to 500 ng.

**Table S1.** Demographic data of the patients and samples.

| **Characteristic** | **N = 30^1^** |
| --- | --- |
| Age | 46 (36, 57) |
| Sex |  |
| F | 10 (33%) |
| M | 20 (67%) |
| WBC (mm^-3^) | 1,790 (1,085, 3,542) |
| Unknown | 4 |
| Platelets (mm^-3^) | 24,000 (16,500, 34,250) |
| Unknown | 4 |
| Hb (g/dL) | 8.85 (7.93, 9.97) |
| Unknown | 4 |
| LDH (U/L) | 313 (245, 461) |
| Unknown | 5 |
| Creatinine (mg/dL) | 0.92 (0.83, 1.00) |
| Unknown | 5 |
| Blasts (%) | 0.87 (0.71, 0.95) |
| Unknown | 4 |
| ^1^ Median (IQR); n (%) | |


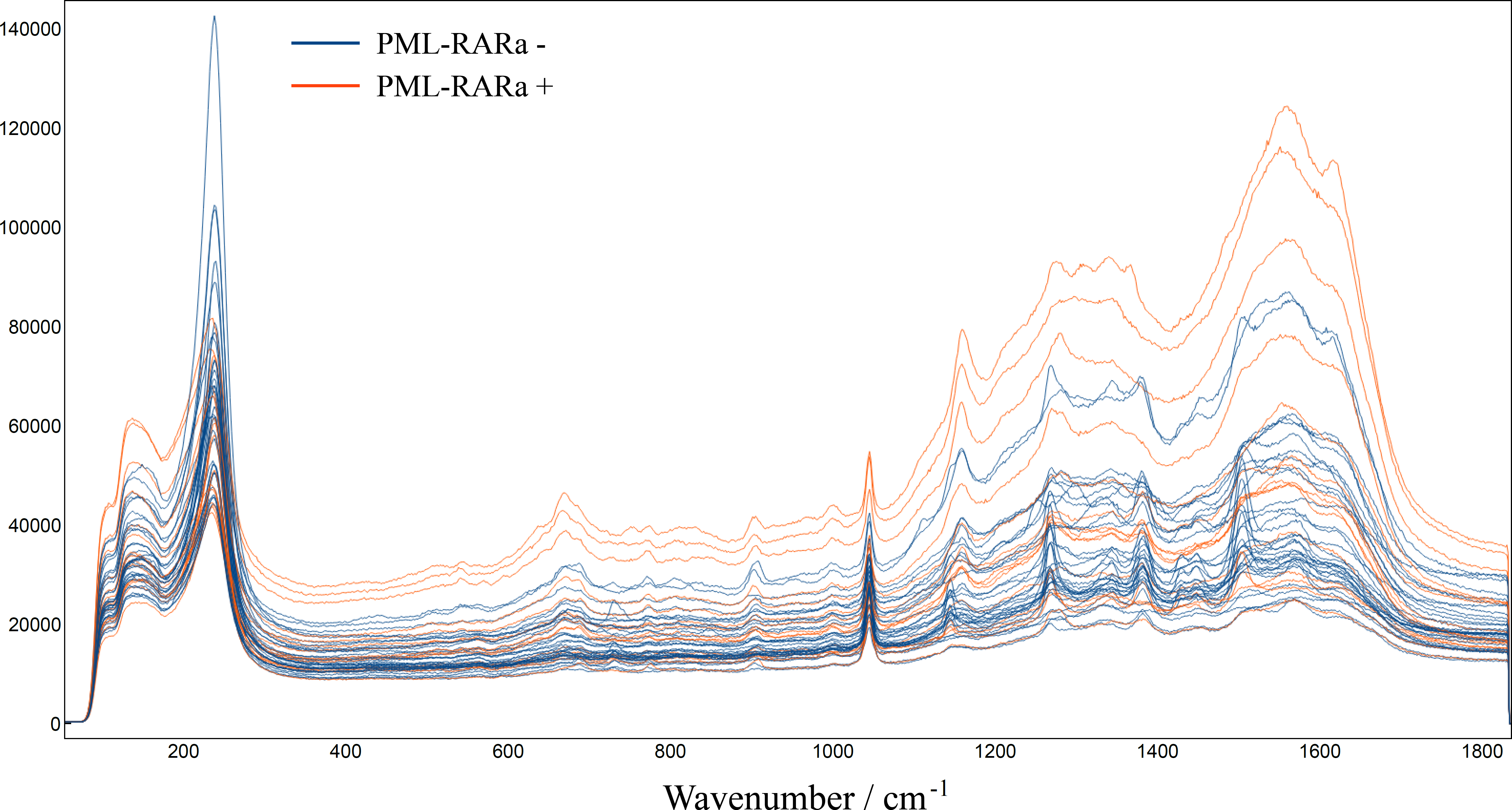


**Figure S4.** The raw SERS spectra of DNA.

**Table S2.** Confusion matrix showing the false positive, false negatives, true positive and false positive samples of the Logistic Regression model.

| Predicted |  | Actual | |
| --- | --- | --- | --- |
|  |  | PML-RARa + | PML-RARa - |
|  | PML-RARa + | 11 | 7 |
|  | PML-RARa - | 4 | 27 |


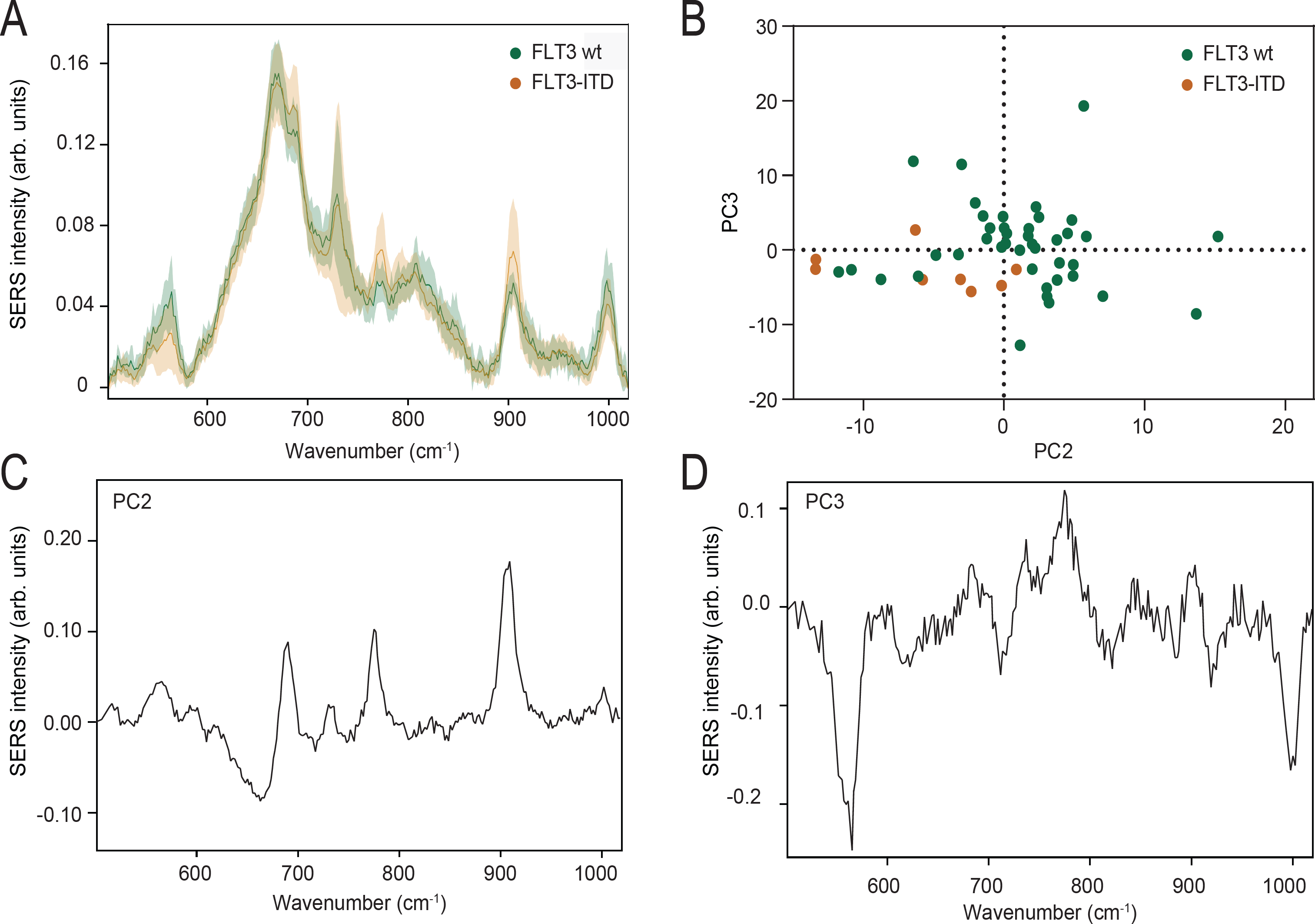


**Figure S5.** A. The average SERS spectra of DNA from patients with *FLT3-ITD* mutations and *FLT3* wildtype (wt). B. The score plots corresponding to principal component (PC) 2 and PC3. C, D. The loading plots of PC3 and PC3, respectively. The dataset consisted of 41 samples in the *FLT3* wildtype group and eight samples in the *FLT3-ITD* group.

**Supplementary discussion**

Among the multitude of strategies that have been evaluated for cancer detection by liquid biopsy, the detection of cancer-associated changes in the DNA methylation pattern is one of the most clinically advanced (Chen et al., 2021). These advancements have been fueled by technologies such as next generation sequencing and DNA methylation profiling arrays which can detect focal changes in DNA methylation down to single CpGs.

DNA methylation primarily occurs at **CpG dinucleotides** in mammals, where a cytosine nucleotide is followed by a guanine nucleotide in the 5' to 3' direction, linked by a phosphate group. This is known as CpG methylation, and it is the most well-studied and prevalent form of DNA methylation in mammalian genomes. The methylation occurs at the 5t^h^ carbon of the cytosine, forming 5-methylcytosine.

There are some 28 million CpG in the human genome, representing less than 2% of the genome. For a review of the epigenetic determinants of cancer, see the excellent review by Baylin and Jones (Baylin & Jones, 2016). The majority of these CpG are scattered randomly in the genome, around 30% are part of the Alu retrotransposons, while some 2% are part of the so called CpG islands, which are regions of 300–3000 base pairs disproportionately enriched for CpGs. The CpG islands are usually found in the 5’ promoter regions of human genes, with around 60% of the genes exhibiting such CpG islands.

Some 80% of the CpGs are methylated in a typical adult somatic cell, except for the CpGs in the CpG islands, which are mostly unmethylated. The unmethylated state of the CpG islands is thought to contribute to the opening of the chromatin, thus allowing gene expression to take place. On the other hand, the methylation of the rest of the CpG contribute functionally to the epigenetic silencing of the transposons.

The epigenetic changes associated with cell transformation involve the reversal of the normal methylation pattern: CpG islands are hypermethylated, while the rest of the CpG are hypomethylated, such that the overall methylation level becomes around 40-60%. The hypermethylation of the CpG islands results in the silencing of many tumor suppressors and contributes functionally to the malignant phenotype. Hypomethylating agents such as azacytidine and decitabine, which trap and inactivate the methyltransferases (Linnekamp et al., 2017), thus reactivating the silenced tumor suppressors, are clinically approved for several hematological malignancies.

In regard to the global hypomethylation, the functional implications are surprisingly poorly understood. Nonetheless, the fact that deletion or reduction of the maintenance DNA methyltransferase, Dnmt1, results in increased mutation rates, aneuploidies, and tumor induction, demonstrates that global hypomethylation does nonetheless represent a driving event and not a passenger one (Gaudet et al., 2003).

Surface-enhanced Raman scattering (SERS) is a method to amplify the Raman signal of molecules that are adsorbed onto the SERS substrates such as gold or silver nanoparticles (Moisoiu, Iancu, et al., 2021). The amplification of the Raman signal depends on the adsorption geometry of the molecules. Several lines of evidence showed that the cancer-associated changes in the DNA methylation pattern promote the preferential assembly of DNA onto the metal gold nanoparticles (Sina et al., 2018), which explains why SERS analysis of DNA can be used for detecting the DNA from cancer cells.

The main challenge in using SERS for the detection of cancer DNA is the interpretation of the spectral changes. In a series of studies concerned with the SERS analysis of DNA from patients with hematological malignancies, we showed that the main spectral changes associated with DNA is a decrease in the SERS band at 1005 cm^-1^ attributed to 5-methylcytosine, which reflects the global hypomethylation of DNA (Moisoiu et al., 2019; Moisoiu, Sas, et al., 2021; Stefancu et al., 2022). On the other hand, the results showed that the DNA from cancer cells exhibits an increase in the intensity of the SERS band at 730 cm^-1^, which suggests that the altered DNA methylation pattern changes the adsorption geometry of the DNA onto the SERS substrate.

The main question of this study was to understand whether a similar pattern of changes in the SERS spectrum of DNA also occurs in the case of APL. The results showed that this is not the case and that the measurable residual disease (MRD) positivity is associated with a decrease in the intensity of the SERS band at 730 cm^-1^ attributed to adenine, while the SERS band at 1005 cm^-1^ was not drastically changed. In addition, we could also see an increase in the SERS band at 770 cm^-1^ attributed to 5-methylcytosine (Ganesh et al., 2020), which in the case of the previous studies had a rather low intensity. One possible explanation for this is the study by Schoofs et al, who showed that DNA from APL cells is not globally hypomethylated but rather hypermethylated (Schoofs et al., 2013). However, it is unclear why the SERS band at 1005 cm^-1^ did not show higher intensities and instead we could see an increase in the SERS band at 770 cm^-1^. Unfortunately, the global DNA methylation levels were not available for the DNA samples used in our study. Nonetheless, quantitative changes in the methylation levels alone are most likely not able to explain the SERS features of the DNA from APL, which probably exhibits qualitative changes in the distribution of methylation marks.

A second question explored in this study regards the effect of the mutation of *FLT3* on the SERS spectral pattern of DNA. The *FLT3* mutations are the most common genetic aberrations found in acute myeloid leukemia (AML) and are present in around 30% of the APLs (Li et al., 2021a). *FLT3* mutations are associated with a poor prognosis, the most common type of mutation being the *FLT3* internal tandem duplication (*FLT3*-ITD) (Levis et al., 2005). Several *FLT3* inhibitors are currently approved in AML (midostaurin, sorafenib, gilteritinib and quizartinib), and are currently explored for relapsed APL (Smith, 2019). Thus, *FLT3* mutations have both prognostic and predictive significance. Among the 30 APL patients included in this study, eight cases exhibited *FLT3*-ITD. Although the number of patients with *FLT3*-ITD was small, we performed nonetheless principal component analysis of the SERS spectra and showed that the spectra corresponding to patients with FLT-ITD APL had the propensity to cluster together in principal components 2 and 3, suggesting that this mutation had a discernible effect on the epigenetic landscape of DNA. The *FLT3* mutations are associated with higher white blood cell counts, with M3v variant morphology, and the bcr3 isoform (Li et al., 2021b), thus demonstrating that *FLT3* mutations profoundly alter the molecular biology of the cells. Although the data presented here is preliminary (and empirical), the possibility to detect *FLT3* mutations by SERS is exciting, because it would bring valuable prognostic and predictive information. However, a detailed understanding of the mechanism behind the effects of *FLT3* mutations on the epigenetic landscape of APL the origin of the observed alterations in the SERS spectra is currently lacking.

An important risk in SERS studies such as these is the use of machine learning algorithms and other AI tools without understanding the molecular origin of the disease-associated spectral changes, which can lead to overfitting and thus to a false increase in the classification accuracy. Overcoming this risk will require careful mechanistic studies as well as a much stringent validation of the models in large, multi-center, prospective studies, which for the moment have not been yet undertaken.

**References**

Baylin, S. B., & Jones, P. A. (2016). Epigenetic Determinants of Cancer. *Cold Spring Harbor Perspectives in Biology*, *8*(9). https://doi.org/10.1101/cshperspect.a019505

Chen, X., Dong, Z., Hubbell, E., Kurtzman, K. N., Oxnard, G. R., Venn, O., Melton, C., Clarke, C. A., Shaknovich, R., Ma, T., Meixiong, G., Seiden, M. V, Klein, E. A., Fung, E. T., & Liu, M. C. (2021). Prognostic Significance of Blood-Based Multi-cancer Detection in Plasma Cell-Free DNA. *Clinical Cancer Research : An Official Journal of the American Association for Cancer Research*, *27*(15), 4221–4229. https://doi.org/10.1158/1078-0432.CCR-21-0417

Ganesh, S., Venkatakrishnan, K., & Tan, B. (2020). Quantum scale organic semiconductors for SERS detection of DNA methylation and gene expression. *Nature Communications*, *11*(1), 1135. https://doi.org/10.1038/s41467-020-14774-3

Gaudet, F., Hodgson, J. G., Eden, A., Jackson-Grusby, L., Dausman, J., Gray, J. W., Leonhardt, H., & Jaenisch, R. (2003). Induction of tumors in mice by genomic hypomethylation. *Science (New York, N.Y.)*, *300*(5618), 489–492. https://doi.org/10.1126/science.1083558

Leopold, N., & Lendl, B. (2003). A New Method for Fast Preparation of Highly Surface-Enhanced Raman Scattering (SERS) Active Silver Colloids at Room Temperature by Reduction of Silver Nitrate with Hydroxylamine Hydrochloride. *The Journal of Physical Chemistry B*, *107*(24), 5723–5727. https://doi.org/10.1021/jp027460u

Levis, M., Murphy, K. M., Pham, R., Kim, K.-T., Stine, A., Li, L., McNiece, I., Smith, B. D., & Small, D. (2005). Internal tandem duplications of the *FLT3* gene are present in leukemia stem cells. *Blood*, *106*(2), 673–680. https://doi.org/10.1182/blood-2004-05-1902

Li, A. Y., Kashanian, S. M., Hambley, B. C., Zacholski, K., Duong, V. H., El Chaer, F., Holtzman, N. G., Gojo, I., Webster, J. A., Norsworthy, K. J., Smith, B. D., DeZern, A. E., Levis, M. J., Baer, M. R., Kamangar, F., Ghiaur, G., & Emadi, A. (2021a). *FLT3*-ITD Allelic Burden and Acute Promyelocytic Leukemia Risk Stratification. *Biology*, *10*(3). https://doi.org/10.3390/biology10030243

Li, A. Y., Kashanian, S. M., Hambley, B. C., Zacholski, K., Duong, V. H., El Chaer, F., Holtzman, N. G., Gojo, I., Webster, J. A., Norsworthy, K. J., Smith, B. D., DeZern, A. E., Levis, M. J., Baer, M. R., Kamangar, F., Ghiaur, G., & Emadi, A. (2021b). *FLT3*-ITD Allelic Burden and Acute Promyelocytic Leukemia Risk Stratification. *Biology*, *10*(3). https://doi.org/10.3390/biology10030243

Linnekamp, J. F., Butter, R., Spijker, R., Medema, J. P., & van Laarhoven, H. W. M. (2017). Clinical and biological effects of demethylating agents on solid tumours - A systematic review. *Cancer Treatment Reviews*, *54*, 10–23. https://doi.org/10.1016/j.ctrv.2017.01.004

Moisoiu, V., Iancu, S. D., Stefancu, A., Moisoiu, T., Pardini, B., Dragomir, M. P., Crisan, N., Avram, L., Crisan, D., Andras, I., Fodor, D., Leopold, L. F., Socaciu, C., Bálint, Z., Tomuleasa, C., Elec, F., & Leopold, N. (2021). SERS liquid biopsy: An emerging tool for medical diagnosis. *Colloids and Surfaces. B, Biointerfaces*, *208*, 112064. https://doi.org/10.1016/j.colsurfb.2021.112064

Moisoiu, V., Sas, V., Stefancu, A., Iancu, S. D., Jurj, A., Pasca, S., Iluta, S., Zimta, A.-A., Tigu, A. B., Teodorescu, P., Turcas, C., Blag, C., Dima, D., Popa, G., Arghirescu, S., Man, S., Colita, A., Leopold, N., & Tomuleasa, C. (2021). SERS-Based Evaluation of the DNA Methylation Pattern Associated With Progression in Clonal Leukemogenesis of Down Syndrome. *Frontiers in Bioengineering and Biotechnology*, *9*, 703268. https://doi.org/10.3389/fbioe.2021.703268

Moisoiu, V., Stefancu, A., Iancu, S. D., Moisoiu, T., Loga, L., Dican, L., Alecsa, C. D., Boros, I., Jurj, A., Dima, D., Bagacean, C., Tetean, R., Burzo, E., Tomuleasa, C., Elec, F., & Leopold, N. (2019). SERS assessment of the cancer-specific methylation pattern of genomic DNA: towards the detection of acute myeloid leukemia in patients undergoing hematopoietic stem cell transplantation. *Analytical and Bioanalytical Chemistry*, *411*(29), 7907–7913. https://doi.org/10.1007/s00216-019-02213-2

Schoofs, T., Rohde, C., Hebestreit, K., Klein, H.-U., Göllner, S., Schulze, I., Lerdrup, M., Dietrich, N., Agrawal-Singh, S., Witten, A., Stoll, M., Lengfelder, E., Hofmann, W.-K., Schlenke, P., Büchner, T., Hansen, K., Berdel, W. E., Rosenbauer, F., Dugas, M., & Müller-Tidow, C. (2013). DNA methylation changes are a late event in acute promyelocytic leukemia and coincide with loss of transcription factor binding. *Blood*, *121*(1), 178–187. https://doi.org/10.1182/blood-2012-08-448860

Sina, A. A. I., Carrascosa, L. G., Liang, Z., Grewal, Y. S., Wardiana, A., Shiddiky, M. J. A., Gardiner, R. A., Samaratunga, H., Gandhi, M. K., Scott, R. J., Korbie, D., & Trau, M. (2018). Epigenetically reprogrammed methylation landscape drives the DNA self-assembly and serves as a universal cancer biomarker. *Nature Communications*, *9*(1), 4915. https://doi.org/10.1038/s41467-018-07214-w

Smith, C. C. (2019). The growing landscape of *FLT3* inhibition in AML. *Hematology. American Society of Hematology. Education Program*, *2019*(1), 539–547. https://doi.org/10.1182/hematology.2019000058

Stefancu, A., Moisoiu, V., Desmirean, M., Iancu, S. D., Tigu, A. B., Petrushev, B., Jurj, A., Cozan, R. G., Budisan, L., Fetica, B., Roman, A., Dobie, G., Turcas, C., Zdrenghea, M., Teodorescu, P., Pasca, S., Piciu, D., Dima, D., Bálint, Z., … Tomuleasa, C. (2022). SERS-based DNA methylation profiling allows the differential diagnosis of malignant lymphadenopathy. *Spectrochimica Acta. Part A, Molecular and Biomolecular Spectroscopy*, *264*, 120216. https://doi.org/10.1016/j.saa.2021.120216

Toplak, M., Read, S. T., Sandt, C., & Borondics, F. (2021). Quasar: Easy Machine Learning for Biospectroscopy. *Cells*, *10*(9). https://doi.org/10.3390/cells10092300
